# Molecular dissection of an immunodominant epitope in K_v_1.2-exclusive autoimmunity

**DOI:** 10.3389/fimmu.2024.1329013

**Published:** 2024-04-11

**Authors:** Ivan Talucci, Friederike A. Arlt, Kai O. Kreissner, Mahoor Nasouti, Anna-Lena Wiessler, Ramona Miske, Swantje Mindorf, Inga Dettmann, Mehrnaz Moniri, Markus Bayer, Peter Broegger Christensen, Ilya Ayzenberg, Andrea Kraft, Matthias Endres, Lars Komorowski, Carmen Villmann, Kathrin Doppler, Harald Prüss, Hans M. Maric

**Affiliations:** ^1^ Rudolf Virchow Center for Integrative and Translational Bioimaging; University of Würzburg, Würzburg, Germany; ^2^ Department of Neurology, University Hospital Würzburg, Würzburg, Germany; ^3^ Department of Neurology and Experimental Neurology, Charité-Universitätsmedizin Berlin, Corporate Member of Freie Universität Berlin, Humboldt-Universität Berlin, Berlin, Germany; ^4^ German Center for Neurodegenerative Diseases (DZNE), Berlin, Germany; ^5^ Institute for Clinical Neurobiology, University of Wuerzburg, Würzburg, Germany; ^6^ Institute for Experimental Immunology, affiliated to EUROIMMUN Medizinische Labordiagnostika AG, Lübeck, Germany; ^7^ Department of Neurology, Aalborg University Hospital, Aalborg, Denmark; ^8^ Department of Neurology, St. Josef-Hospital, Ruhr-University Bochum, Bochum, Germany; ^9^ Department of Neurology, Hospital Martha-Maria, Halle, Germany; ^10^ Klinik und Hochschulambulanz für Neurologie, Charité-Universitätsmedizin, Berlin, Germany; ^11^ Center for Stroke Research, Berlin, Germany; ^12^ German Centre for Cardiovascular Research (DZHK), Berlin, Germany; ^13^ German Center for Mental Health (DZPG), Berlin, Germany

**Keywords:** autoantibodies, K_v_ channel, KCNA2, autoimmune encephalitis, epitope mapping, immunodominant antigen, dementia, critical

## Abstract

**Introduction:**

Subgroups of autoantibodies directed against voltage-gated potassium channel (K_v_) complex components have been associated with immunotherapy-responsive clinical syndromes. The high prevalence and the role of autoantibodies directly binding K_v_ remain, however, controversial. Our objective was to determine K_v_ autoantibody binding requirements and to clarify their contribution to the observed immune response.

**Methods:**

Binding epitopes were studied in sera (n = 36) and cerebrospinal fluid (CSF) (n = 12) from a patient cohort positive for K_v_1.2 but negative for 32 common neurological autoantigens and controls (sera n = 18 and CSF n = 5) by phospho and deep mutational scans. Autoantibody specificity and contribution to the observed immune response were resolved on recombinant cells, cerebellum slices, and nerve fibers.

**Results:**

83% of the patients (30/36) within the studied cohort shared one out of the two major binding epitopes with K_v_1.2-3 reactivity. Eleven percent (4/36) of the serum samples showed no binding. Fingerprinting resolved close to identical sequence requirements for both shared epitopes. K_v_ autoantibody response is directed against juxtaparanodal regions in peripheral nerves and the axon initial segment in central nervous system neurons and exclusively mediated by the shared epitopes.

**Discussion:**

Systematic mapping revealed two shared autoimmune responses, with one dominant K_v_1.2-3 autoantibody epitope being unexpectedly prevalent. The conservation of the molecular binding requirements among these patients indicates a uniform autoantibody repertoire with monospecific reactivity. The enhanced sensitivity of the epitope-based (10/12) compared with that of the cell-based detection (7/12) highlights its use for detection. The determined immunodominant epitope is also the primary immune response visible in tissue, suggesting a diagnostic significance and a specific value for routine screening.

## Highlights

- K_v_ autoantibodies are prevalent but of controversial diagnostic significance.
- K_v_-exclusive autoimmunity in 30 patients is characterized by a uniform and monospecific autoantibody repertoire.
- A single immunodominant Kv1.2/1.3 autoantibody epitope has been identified.
- The dominant epitope manifests as the only immune response in all tested tissues and cells.

## Introduction

Neurological diseases associated with autoantibodies are increasingly recognized as new medical entities (1) but frequently remain to be fully resolved at the molecular level. The identification of exact underlying disease-defining autoantibody epitopes is critical for understanding and addressing the root cause of these clinical entities. Advanced peptide microarray technologies demonstrated a remarkable success in resolving comprehensive linear epitope landscapes from raw patient samples (2, 3). Here, we used a peptide microarray–based readout (4) for analyzing the largest so far studied cohort with K_v_1.2-exclusive immune response in molecular detail.

Autoantibodies directed against K_v_1 channel complexes have been identified in several neurological diseases, including autoimmune encephalitis, limbic encephalitis, and Morvan’s syndrome (5, 6). Within this group, anti-leucine-rich glioma inactivated 1 (LGI1) and anti-Contactin-associated protein-like 2 (Caspr2) autoantibodies are among the most prevalent, and these have been associated with clinical syndromes that are immunotherapy-responsive (7, 8). Previous studies also identified highly prevalent intracellular binding of K_v_1 antibodies, many of which are targeting intracellular epitopes. Only a fraction of the intracellular positive K_v_1 patients (27%) showed a sustained immunotherapy benefit (7). The subgroup of K_v_1 channels play a critical role in regulating neurotransmission in both the central and peripheral nervous system by controlling the flux of potassium ions from the neuron during the action potential. K_v_1.3 was related not only to astrocyte activation in experimental autoimmune encephalitis but also to CD4^+^ T-cell differentiation during inflammatory immune–mediated disease (9). Pharmacological and knockout blockade of this channel has been shown to suppress these functions (10, 11). K_v_1.2 knockout mice show seizures in early developmental stages (12). Furthermore, K_v_1.2 dysfunction has been associated not only with epilepsy (13–15) and developmental disorders (16, 17) but also with multiple sclerosis (18) and neuroinflammatory disorders due to its crucial role in T-lymphocytes (19). The role of K_v_1.2 in autoimmune disorders remains to be fully explored (20). K_v_1.2 autoantibodies were shown to exacerbate an epileptic phenotype in rodent models (21), and cases of K_v_1 autoantibody–associated limbic encephalitis were reported to be immunotherapy-sensitive (22). *In vitro* evidence suggests that autoantibodies can potentially reach their intracellular epitopes through Fc receptor–mediated internalization (23, 24), consequently leading to a smoldering autoimmunity. The high prevalence and the functional role of anti–K_v_1.2 autoantibodies *in vivo* and their association with specific clinical phenotypes are currently undefined, thereby potentially limiting diagnostic and therapeutic options. This is in part due to the lack of molecular knowledge on the involved epitopes and their contribution to the observed immune response.

Here, we report the screening, mapping, and validation of two K_v_1.2 epitopes in 32 patients, thereby providing detailed molecular information on K_v_1.2 autoimmunity and a basis for the development of diagnostic approaches.

## Methods

### Collection of human sera and CSFs

The study comprised sera from 18 controls and 36 patients, along with cerebrospinal fluid (CSF) from five controls and 12 patients. The patients exhibited diverse neuropsychiatric disease phenotypes, including neurodegenerative disorders and autoimmune encephalitis. Two patients (ID 20 and 21), included in this work, have been previously published (20); their CSF samples were not available. Data of all patients were collected at the Charité, Berlin, and all participants gave an informed written consent. All patient sera underwent cell-based screening at Euroimmun (EUROIMMUN Medizinische Labordiagnostika AG). Immunoglobulin G (IgG) detection was conducted using a biochip array with acetone-fixed recombinant human embryonic kidney 293 (HEK293) cells separately expressing the following autoantigens: Aquaporin-4 (AQP4), Rho GTPase activating protein 26 (ARHGAP26), sodium-potassium ATPase catalytic subunit alpha-3 (ATP1A3), Contactin-1 (CNTN1), collapsin response mediator protein 5 (CRMP5 or CV2), Carbonic Anhydrase-Related Protein VIII (CARP VIII), contactin-associated protein-like 2 (CASPR2), Tr/Delta/Notch-like epidermal growth factor-related receptor (DNER/Tr), Dipeptidyl-Peptidase-like Protein-6 (DPPX), Flotilin 1/2, γ-aminobutyric acid type B (GABA_B_) receptor, glutamic acid decarboxylase 65 (GAD65), γ-aminobutyric acid type A (GABA_A_)receptor, glycine receptor, glutamate receptors [types α-Amino-3-hydroxy-5-methylisoxazole-4-propionic acid (AMPA) receptor, Glutamate Receptor ionotropic delta-2 (GLURD2), Metabotropic glutamate receptor 1 (mGluR1), Metabotropic glutamate receptor 5 (MGluR5), N-methyl-D-aspartate (NMDA)], Homer 3, Hu, Inositol 1,4,5-Trisphosphate Receptor Type 1 (ITPR1), IgLON family member 5 (IgLON5), K_v_1.2, leucine-rich glioma inactivated 1 (LGI1), myelin oligodendrocyte glycoprotein (MOG), Neurochondrin (NCDN), Neurofascin 155 (NF155), Neurofascin 186 (NF186), Paraneoplastic antigen Ma2 (PNMA2), recoverin, Ri paraneoplastic antigen, Seizure Related 6 Homolog Like 2 (Sez6L2), Yo paraneoplastic antigen, Zic Family Member 4 (ZIC4). The initial laboratory pre-screening encompassed a cohort of 96 healthy controls, from which four individuals exhibited positive K_v_1.2 autoantibodies (4.2% positive healthy donors). Subsequently, these samples were subjected to detailed peptide microarray–based evaluation.

### Microarray synthesis and quality control

The complete K_v_ sequences (UniProtKB: Q09470, P16389, P22001, and P22459) were displayed in microarray format as 15-mer overlapping peptide libraries. Peptide arrays were synthesized using µSPOT (25), a SPOT-based (26) synthesis approach. In brief, custom-prepared discs containing 9-fluorenylmethyloxycarbonyl(Fmoc)-β-alanine linkers (average loading: 130 nmol/disc, 4 mm in diameter) were loaded in a MultiPep rSi robot (CEM GmbH, Kamp-Lintford, Germany) together natural amino acid (AA) building blocks and phospho-building blocks from IRIS (IRIS Biotech GmbH, Marktredwitz, Germany) and the freshly prepared reagents. Synthesis was carried out by deprotecting the Fmoc-group using 20% piperidine in dimethylformamide (DMF). Peptide chains were elongated using a coupling solution consisting of aAs (0.5 M) with Oxyma (1 M) and diisopropylmethanediimine (1 M) in DMF (1:1:1). Coupling steps were carried out three times (30 min), followed by capping (4% acetic anhydride in DMF). Discs were transferred into 96–deep-well plates for the workup. Side chains were deprotected using 90% trifluoracetic acid (TFA), 2% dichloromethane (DCM), 5% H_2_O, and 3% triisopropylsilane (TIPS) (150 μL per well) for 1 h at room temperature (RT). Afterwards, the deprotection solution was removed, and the discs were solubilized overnight (ON) at RT while shaking, using a solvation mixture containing 88.5% TFA, 4% trifluoromethanesulfonic acid, 5% H_2_O, and 2.5% TIPS (250 μL per well). The resulting peptide-cellulose conjugates were precipitated with ice-cold ether (700 μL per well) and spin down at 2,000×g for 10 min at 4°C, followed by two additional washes of the formed pellet with ice-cold ether. The resulting pellets were dissolved in DMSO (250 μL per well).

Liquid chromatography–mass spectrometry (LC-MS) (27) was carried out using peptide quality controls that were cleaved from the solid support. To ensure cleavage, a Rink amide linker (Iris) suitable for Solid Phase Peptide synthesis (SPPS) on cellulose support was introduced during the first coupling cycle. In an acidic environment, the quality controls were cleaved off the solid support. To isolate the quality controls, 150 µL of the supernatant was transferred to 1.5-mL reaction tubes, followed by the addition of 700 µL of diethyl ether. The samples were then vortexed, and the peptides were allowed to precipitate by incubation at −20°C overnight. After centrifugation at 13,300×g and 4°C for 10 min, the supernatant was discarded, and 500 µL of diethyl ether was added. The mixture was vortexed and centrifuged for 10 min, and the supernatant was decanted. This process was repeated twice, and the peptides were left to dry for 60 min. Finally, the Rink amides were dissolved in 50 µL of 50% acetonitrile and 0.1% formic acid (v/v) and vortexed briefly before centrifugation at 13,300×g and RT. For analysis, the quality controls were diluted 1:3 and analyzed via LC-MS (Agilent technologies).

### Microarray printing and binding assay

Peptide-cellulose conjugate (PCC) solutions were mixed 2:1 with saline-sodium citrate buffer [150 mM NaCl and 15 mM trisodium citrate (pH 7.0)] and transferred to a 384-well plate. For transfer of the PCC solutions to white-coated CelluSpot blank slides (76 mm × 26 mm, Intavis AG Peptide Services GmbH and Co. KG), a SlideSpotter (CEM GmbH) was used. After completion of the printing procedure, slides were left to dry overnight.

The microarray slides were blocked for 60 min in 5% (w/v) skimmed milk powder (Carl Roth) 0.05% Tween 20 phosphate-buffered saline [PBS; 137 mM NaCl, 2.7 mM KCl, 10 mM Na_2_HPO_4_, and 1.8 mM KH_2_PO_4_ (pH7.4)]. After blocking, the slides were incubated for 30 min with either positive and negative serum or cerebrospinal fluid (dilution ranging from 1:300 to 1:3,000) or K_v_1.2 monoclonal (NeuroMab clone: K14/16 at 0.5 µg/mL) in the blocking buffer and then washed three times with PBS 0.05% and Tween 20 for 1 min. IgG antibodies were detected using goat anti-human IgG-Horseradish peroxidase (HRP) (Thermo Fisher, Cat. No. 31410; 1:2,500) or goat anti-mouse IgG-HRP (Thermo Fisher, Cat. No. 31430; 1:5000). The chemiluminescence readout was detected with an Azure imaging system c400 (lowest sensitivity, exposure time of 60 s for serum and of 30 s for monoclonal) using SuperSignal West Femto maximum sensitive substrate (Thermo Scientific, GmbH, Schwerte, Germany). Microarray binding intensities were quantified using the MicroArray Rastering Tool – MARTin (28). Neutralization assays were performed by pre-incubating the sera in neutralizing solution, containing cleavable peptides or buffer for 30 min. The binding assay followed in the same manner.

### Recombinant expression of K_v_1 in human embryonic kidney 293 cells

K_v_1.1, K_v_1.2, and K_v_1.6 were expressed in HEK293 cells, following a previously described protocol (Miske et al., 2023). To summarize, genomic DNA was extracted from HEK293 cells and utilized as a template for K_v_1.2 coding sequence amplification via polymerase chain reaction (PCR). Respective DNA oligonucleotides were employed to introduce the required enzyme restriction sites (**Table 1**). Indicated enzymes were used to digest the resulting PCR fragments and subsequently ligated with *Nco*I/*Xho*I-linearized pTriEx-1 (Merck). Prior to transfection, HEK293 cells were plated on sterile poly-L-lysine–treated coverslips. Transient expression of K_v_1-encoded proteins was accomplished through Polyethylenimine (PEI)-mediated transfection (PEI 25K™) following the manufacturer’s instructions (Polysciences, Europe). After 48 h of transfection, cells were fixed, permeabilized with acetone, and rinsed with PBS before conducting the immunofluorescence described below.

**Table 1:** DNA oligonucleotide primers for PCR amplification of K_v_1.2, K_v_1.1, and K_v_1.6.

Protein	Restriction sites	DNA oligonucleotide sequence (5′-3′)
K_v_1.2	NcoI XhoI	F: ATTCCATGGCAGTGGCCACCGGAGACCCAGCAGACGAG R: TATCTCGAGTCAGACATCAGTTAACATTTTGGTAATATTCAC
K_v_1.1	Eco31I XhoI	F: ATTAGGTCTCACATGACGGTGATGTCTGGGGAGAACGTGGA R: TATCTCGAGTTAAACATCGGTCAGTAGCTTGCTCTTATTAACG
K_v_1.6	PagI XhoI	F: ATATCATGAGATCGGAGAAATCCCTTACGCTGGCG R: TATCTCGAGTCAGACCTCCGTGAGCATTCTTTTCTCTG

F, forward primer; R, reverse primer.

### Mouse brain and sciatic nerve processing

All animal procedures were approved by the Landesamt für Gesundheit und Soziales (LaGeSo) Berlin, Germany (approval numbers T-CH 0009/22), and conducted in compliance with the German and international guidelines for care and humane use of animals. Unfixed brain processing and sciatic nerve teased fiber preparations were performed as previously described (29). In brief, male C57BL/6 mice were used at an age of 10–12 weeks. Unfixed brains were dissected and frozen in 2-methylbutan. Cryostat-cut 20-µm sections were mounted on glass slides and used for tissue-based immunofluorescence. Murine sciatic nerves were dissected from hind limbs, fixed in 4% paraformaldehyde, and washed in PBS. The epineurium was removed prior to teasing. Teased fibers were air-dried and stored at −20°C until further usage.

### Cell- and tissue-based immunofluorescence binding assays

Slides and cells were post-fixed with acetone and washed with PBS. Serum, CSF, and/or commercial K_v_1.1 (Abcam ab65790), K_v_1.2 (K14/16 Neuromab, supplier Antibodies Incorporated 75-008), and K_v_1.6 antibody (Antibodies Incorporated 75-012) incubation was done overnight at +4°C in a dilution of 1:300 (serum), 1:2 (CSF), and 1:200 (commercial) in PBS-T (containing 0.2% Tween 20 and 0.1% Triton X). Secondary antibodies against human IgG (Alexa488-labeled Dianova, 109-545-003; and Cy3-labeled Dianova, 109-165-003), mouse IgG (Alexa549-labeled goat anti-mouse, Jackson Research, 115-585-03; and Alexa-488-labeled, Dianova, 115-546-003) and rabbit IgG (Alexa488-labeled, Dianova, 111-546-003) were added in a dilution of 1:1,000 in PBS-T for 1 h at RT. Cell nuclei were stained using 4',6-diamidino-2-phenylindole (DAPI). Coverslips and glass slides were mounted in Fluoroshield and visualized using a widefield fluorescence microscope (LeicaSPE).

### Cell and tissue neutralization of anti-K_v_ sera

Neutralization assays were adapted from established protocols (Miske et al., 2023). In brief, peptides were solubilized in PBS at a final concentration of 1 mg/mL. Neutralization assays were performed on transfected HEK293 cells and on mouse tissues. Sera were diluted in PBS-T to the abovementioned concentrations. The peptide antigen was added in a dilution of 1:10. Sera and peptides were thereby pre-incubated for 1 h at RT and subsequently added to slides and cells incubating for 1 h at RT. After washing with PBS-T, cells and slides were stained with secondary Alexa488-labeled antibodies for 30 min at RT. After washing with PBS-T, cell nuclei were stained with DAPI. Co-staining with the commercial K_v_1.2 antibody (K14/16 NeuroMab) and secondary Alexa594-labeled antibody was accomplished through sequential staining rounds, thereby avoiding potential cross-reactivity of secondary antibodies to primary mouse or human antibodies.

### Data availability

Data are available upon reasonable request to qualified investigators for the purposes of replicating procedures and results.

## Results and discussion

### Patient samples

Screening, mapping, and validation of K_v_1.2 epitopes was based on 36 samples from patients (74.3% men, median disease onset age of 65, median high sera titer of 1:1,000, with four out of the 96 positive healthy controls positive in this readout) positive for K_v_1.2 but negative for 33 common neurological autoantigens in cell-based assays. Patients showed heterogeneous neuropsychiatric phenotypes ranging from dementia, to epilepsy, autoimmune encephalitis, ischemic strokes, and peripheral neuropathies. Patient ID 19 had anti-AP3B2 IgG (titer 1:32) and patient ID 30 anti-NMDAR IgM (1:1,000) autoantibodies in serum; in the rest of the cohort, no other co-existing antibodies were detected.

### Single–amino acid resolution mapping of two immunodominant K_v_1.2 epitopes

**Figure 1:**
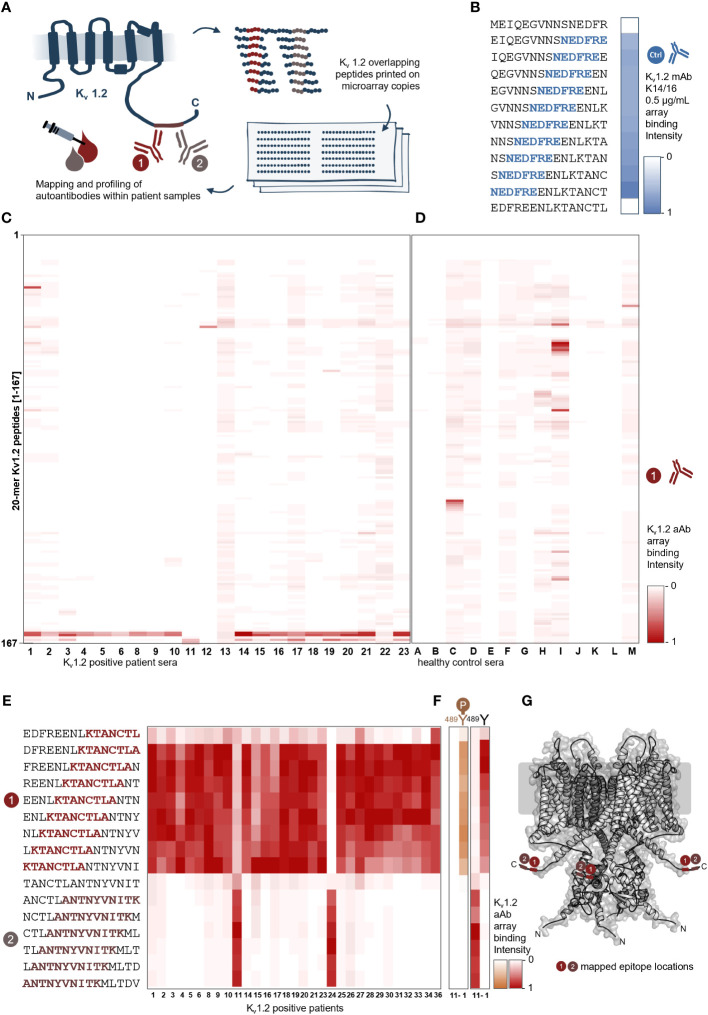
Peptide microarray screening of anti–K_v_1.2-positive patient samples reveals two shared binding epitopes. **(A)** Scheme of the K_v_1.2 microarray display. K_v_1.2 complete primary sequence was displayed in microarray format as 20-mer peptides with 17-residue overlap, and autoantibody binding was detected using goat anti-human IgG coupled to HRP for chemiluminescence readout. **(B)** Array validation using a commercial antibody. The microarray reports the residues *NEDFRE* as the core motif for Anti-mouse K_v_1.2 (NeuroMab clone K14/16) that was raised against immunogen (Fusion K_v_1.2 residues: 428–499) and previously mapped for the following sequence *EGVNNSNEDFREENLKTA*. **(C)** Identification of two distinct epitopes from sera. Epitope mapping of 22 patient sera. The sera reactivity of 22 positive samples and **(D)** 13 negative samples over 161 peptides were analyzed and plotted as a heat map over the most prominent peptide binder [0-1]; each rectangle corresponds to a single patient sample reactivity. Two main epitopes were identified between the peptides 156–161. **(E)** Single–amino acid mapping resolves the two minimal motifs. A 15-mer library revealed a shortest motif for E1 (*KTANCTLA*) and E2 (*ANTNYVNITK*). **(F)** Phosphorylation dependency of the two autoantibody epitopes. Here, two different samples (left: sera 11; right: sera 1) were tested on unphosphorylated and phosphorylated Y-489 peptides. E2 from patient 11 is negatively affected by phosphorylation; in contrast, E1 from patient 1 is not affected. **(G)** Visualization of the identified core motifs. Cartoon model of the K_v_1.2 tetramer highlighting the two intracellular epitopes.

To identify anti-K_v_1.2 epitopes, the qualified patient samples were screened in peptide microarray format. Here, the entire primary sequence of K_v_1.2 was displayed in the form of 20-mer peptides with 17-residue overlap (**Figure 1A**). First, the array approach was validated using the commercial anti-mouse K_v_1.2 (NeuroMab clone K14/16) antibody. The microarray defined the sequence *NEDFRE* as the core motif (**Figure 1B**), thereby recapitulating the expected (^463^ *EGVNNSNEDFREENLKTA* ^480^) epitope. Next, 22 seropositive (**Figure 1C**) and 13 seronegative (control) (**Figure 1D**) sera were probed on the K_v_1.2 array. Here, autoantibody binding was detected using anti-human IgG coupled to HRP for chemiluminescence detection. The screening identified two prominent intracellular (**Figure 1E**) epitopes: *Epitope 1* (E1) (^469^ *NEDFREENLKTANCTLA^485^*) and *Epitope 2* (E2) (^481^ *NCTLANTNYVNITK^495^*). Notably, none of the tested sera shared both epitopes. An additional array library with a single-AA shift of 15-mer peptides (**Figure 1F**) defined ^478^ *KTANCTLA* ^485^ as the E1 core motif, which was shared among 30 patients (Serum IDs 1–6, 8–10, 14–21, 23–34, and 36) and ^485^ *ANTNYVNITK* ^495^ as the minimal required E2 core motif, which was shared by two patients (Serum IDs 11 and 24) (**Table 2**).

**Table 2:** Serum and cerebrospinal fluid peptide microarray samples used in this study.

Patient ID	Serum - array	CSF - array	Ctrl ID	Serum/ CSF- array
1	E1	E1	A	–
2	E1	E1	B	–
3	E1	E1	C	–
4	E1	E1	D	–
5	E1	E1	E	–
6	E1	n/a	F	–
8	E1	n/a	G	–
9	E1	n/a	H	–
10	E1	n/a	I	–
11	E2	n/a	J	–
12	–	–	K	–
13	–	n/a	L	–
14	E1	n/a	M	–
15	E1	n/a	N	–
16	E1	n/a	O	E1
17	E1	E1	P	E1
18	E1	n/a	Q	E1
19	E1	n/a	DC1	–
20	E1	n/a	DC2	–
21	E1	n/a	DC3	–
22	E1	E1	DC4	–
23	E1	E1	DC5	–
24	E2	n/a		
25	E1	E1		
26	E1	n/a		
27	E1	n/a		
28	E1	n/a		
29	E1	n/a		
30	E1	n/a		
31	E1	n/a		
32	E1	n/a		
33	E1	n/a		
34	E1	n/a		
35	–	–		
36	E1	E1		

ID, identity; CSF, cerebrospinal fluid; na, not available; (−), negative; E1, epitope 1; E2, epitope 2. Sample IDs: K_v_1.2-positive sera (1–6, 8–28, 30–37), control sera [A–Q], K_v_1.2-positive CSF (1–5, 12, 17, 22, 23, 25, 36, 37), control CSF [DC1–5]. Note: ID 12 resulted negative from the arrays and positive for K_v_1.1 in cell-based assay (data not shown).

Remarkably, the identified epitopes both overlap and include a validated phosphorylation site (^389^Tyr; PhosphoSitePlus: P16389). In order to delineate the differences in the mode of binding between these two antibodies and specifically their dependency on phosphorylation, we next probed the corresponding phospho-tyrosine K_v_1.2 library. Whereas autoantibody binding of E1 was not affected, E2 binding was completely abolished upon phosphorylation (**Figure 1G**).

### Autoantibody binding profiles hint toward a common molecular motif

**Figure 2:**
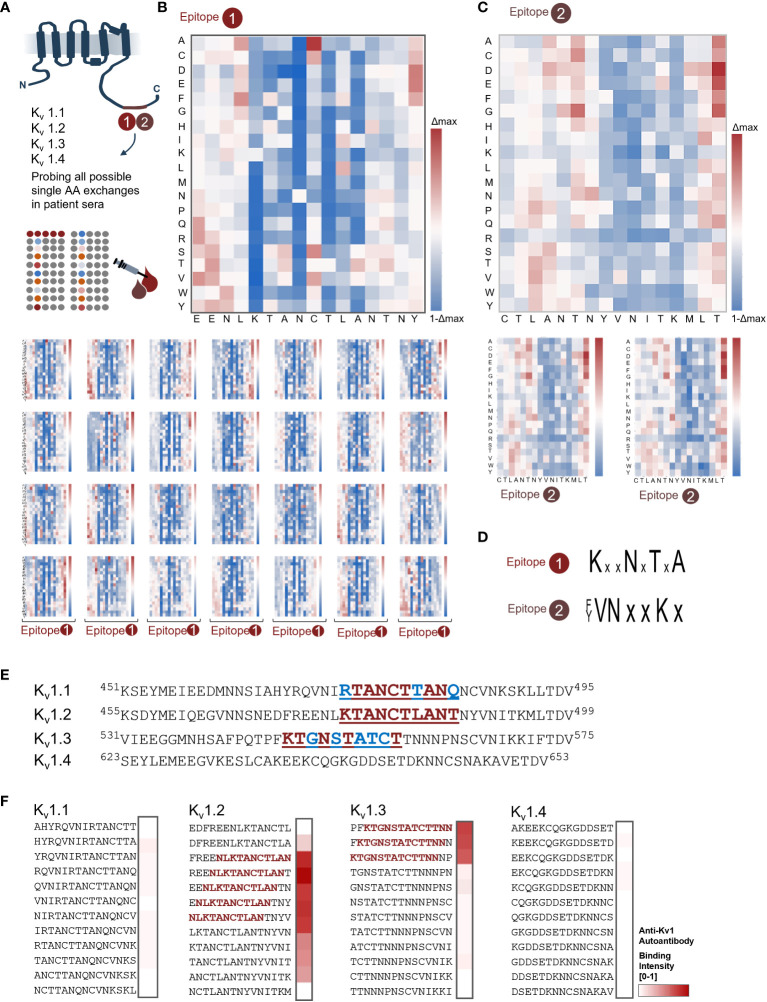
Deep mutational scans reveal common binding profiles for both shared epitopes. **(A)** Scheme of the K_v_1.2 microarray profiling of E1 and E2 core motifs. From both epitopes, each possible positional substitution was generated (342 variants for each epitope) and printed in microarray format. **(B)** Fingerprint analysis for sera containing autoantibody in the E1 group. Heat map overview of the major epitope fingerprint from serum 1; the wt sequence was sequentially scanned from N to C terminal by exchanging each position into each proteinogenic amino acid. Subsequently, the residue binding contribution was depicted in blue-white-red shades, where white corresponds to no variation over the wt [1], blue shades depict a loss, and red shades a gain of binding intensity. The most conserved residues for E1 were K^478^, N^481^, and T^483^. **(C)** Fingerprint analysis revealed a shared profile in the E2 group. General overview of the binding profile for E2 (patient 11) shows matching conserved residues. Here, Y^489^, V^490^, N^491^, and K^494^ were the most conserved residues. **(D)** Recapitulated minimal motifs for E1 and E2. **(E)** Alignment of K_v_1 channels shows high homology for the 1.1, 1.2, and 1.3 subunits. Residues “PQTP” on K_v_1.3 are the known Cortactin interface, where the “TDV” sequence is a known PDZ-binding interface **(F)** C-terminal K_v_1 peptides display reveals novel co-occurring autoantigen “sub-scenario” for E1. Combined K_v_1.1, K_v_1.2, K_v_1.3, and K_v_1.4 peptides were probed with patient 1 sera from the E1 group. In addition to the already mapped K_v_1.2 epitope, the K_v_1.3 peptides showed strong binding as depicted in the heat map. In line with the previous fingerprinting analysis, the R to K exchange in-between K_v_1.1 and K_v_1.2/1.3 abolishes binding.

To further resolve the binding requirements for autoantibodies, we conducted a deep mutational scan of the newly defined minimal binding epitopes. These libraries comprised all possible single-point variants of 18-mer peptides that harbored the minimal core motifs. The subsequent fingerprint analysis of the resulting 2 × 342 peptide variants (**Figure 2A**) confirmed both the previously defined minimal core motifs (**Figure 1C**) and the impact of the Tyr^489^ phosphorylation (**Figure 1G**).

Notably, for E1, all patient samples displayed a seemingly identical binding requirement for their K_v_1.2 autoantibodies (**Figures 2B, C**). More precisely, within the mapped core motif ^78^ *KTANCTLA* ^485^ (**Figure 1C**) residues, ^478^Lys, ^481^Asn, and ^483^Thr were characterized by strict conservation with no tolerance toward any AA exchange (**Figure 2B**) for all tested patients (**Figure 2C**). For E2, both patients were fingerprinted (**Figure 2D**). Here, the fine-mapped core motif ^485^ *ANTNYVNITK* ^495^ (**Figure 1C**) was also recapitulated by both patients in the same way. In addition to the mapping, the fingerprint further highlights a strong conservation of the C-terminal part of the core motif ^489^Tyr to ^495^Lys and strict conservation of ^490^Val, ^491^Asn, and ^494^Lys. In line with the analysis of the phosphorylated peptides (**Figure 1G**), the phophomimetic exchange of Tyr^489^ with Glu resulted in complete loss of autoantibody recognition, thereby confirming our previous finding of orthogonal recognition of this Tyrosine depending on its phosphorylation status. In summary, the profiling highlights and substantiates a shared autoimmune response toward two distinct K_v_1.2 epitopes. The unexpectedly high similarities in the relative binding responses within the patient cohort and the strict conservation of identical residues suggest a homogenous autoantibody repertoire within the tested patient group and further hint toward a shared molecular origin of the observed epitopes.

The resolved binding profiles prompted us to next explore K_v_-isoform specificity of the immunodominant antibody. Comparing the alignment of K_v_ subfamily members 1.1 to 1.4 with the previous binding requirements highlights the conservation of critical residues within these four subfamily members (**Figure 2E**), specifically between K_v_1.1, K_v_1.2, and K_v_1.3. We therefore displayed and probed overlapping C-terminal peptides of all four members of this subfamily in microarray format and found that autoantibody binding to E1 is maintained within K_v_1.2 and K_v_1.3 but not K_v_1.1 and K_v_1.4 (**Figure 2F**). The additional binding capacity for K_v_1.3 is in line with the previously resolved binding requirements of E1 (**Figure 2C**). Vice versa, the lack of binding of K_v_1.1 that shares high homology in this region highlights the need for experimental validation of putative binders.

### Array detection complements cell-based assay

**Figure 3:**
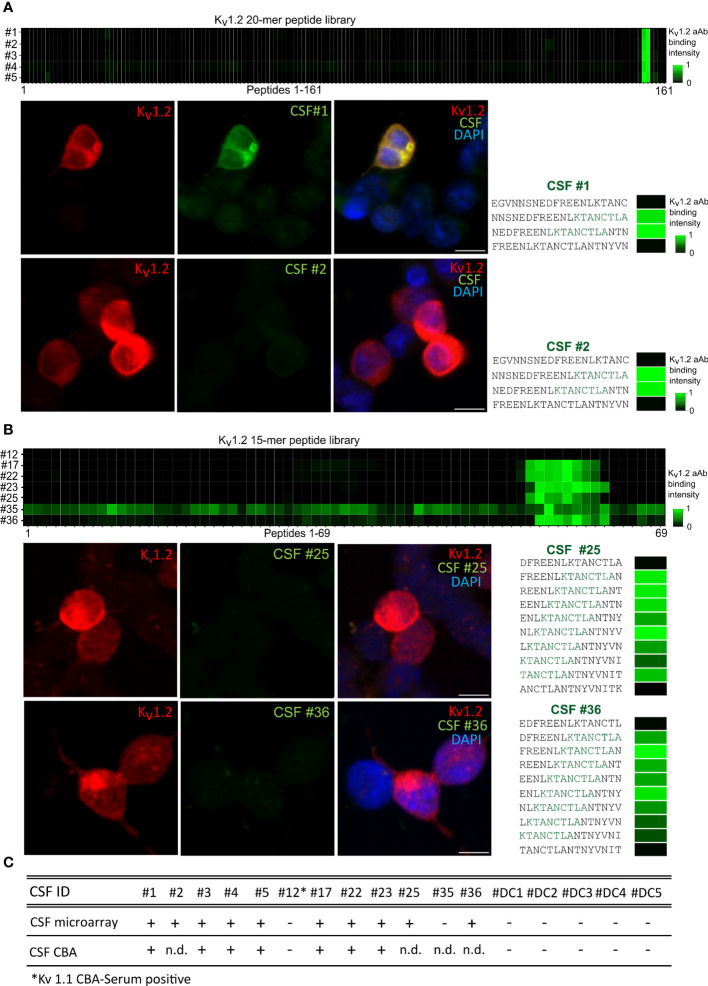
Higher sensitivity of the microarray over cell-based detection for CSF anti-K_v_1.2. **(A)** K_v_1.2 autoantibody detection within CSF by CBA and 20-mer peptides in array format. Top: Cerebrospinal fluid autoantibody binding of six positive samples on 161 K_v_1.2 peptides. E1 has been detected between the overlapping peptides 156–157. Bottom: Comparison of HEK293 cell K_v_ 1.2 binding assay and 20-mer peptide array for CSF 1 and 2. **(B)** K_v_1.2 autoantibody detection within CSF by CBA and 15-mer peptides in array format. Core binding motif resolved for seven additional CSF-positive patients, binding observed between the K_v_1.2 peptides 55–62. Comparison of HEK293 cell K_v_1.2 binding assay and 15-mer peptide array for patients 25 and 36. **(C)** Overview of the K_v_1.2 autoantibody detection within CSF. CSFs from patients 2, 25, and 36 tested positive in microarray but negative in CBA under the conditions tested. Patient 12 was tested positive for K_v_1.1 and K_v_1.2 autoantibodies in serum CBA. n.d., non detected.

To resolve the predictive value of the specific epitope signals, we correlated seropositivity with the observed disease phenotype. To this end, the Euroimmun pre-screening determined 4% of the samples from healthy individuals as anti–K_v_1.2-positive (four out of the 96 samples, data not shown). Our microarray readout recapitulated the reactivity and deep mutational scan analysis and attributed it toward E1, thus suggesting a similar broad occurrence (4%) of these mono-reactive autoantibodies within healthy individuals (**Supplementary Figure 1**). In this light, we focused on the CSF samples obtained from 12 patients exploring both the diagnostic value and the sensitivity of the array screening in comparison to cell-based screening and its dependence of key parameters of the peptide display (**Figure 3**). The CSF of first five patients was analyzed with longer peptides and larger offset (20-mers, offset of 3) (**Figure 3A**), and CSF from seven patients was screened using shorter peptides with shorter offset (15-mers, offset of 1) (**Figure 3B**) (**Table 2**). Complementation of the array-based screening with cell-based screens showed equal in several cases even superior sensitivity for both display variants (7/12 CBA and 10/12 array detected patients) (**Figure 3C**). Specifically, CSFs from patients 2, 25, and 36 were tested positive in microarray and negative in CBA under the tested conditions. In addition, patient 12, negative from the arrays, resulted positive for K_v_1.2 and K_v_1.1 in cell-based assay (data not shown), potentially bearing a conformational epitope. Importantly, the CBA-determined positivity showed an association with cognitive impairment (30). Here, the array showed improved sensitivity over the CBA-based assay. We therefore conclude that, for K_v_ autoimmunity patients, clinicians should consider combining array- and CBA-based testing for autoantibody confirmation, when an autoimmune pathogenesis is suspected.

### The identified K_v_1.2 epitopes are the exclusive mediators of the observed immune response

**Figure 4:**
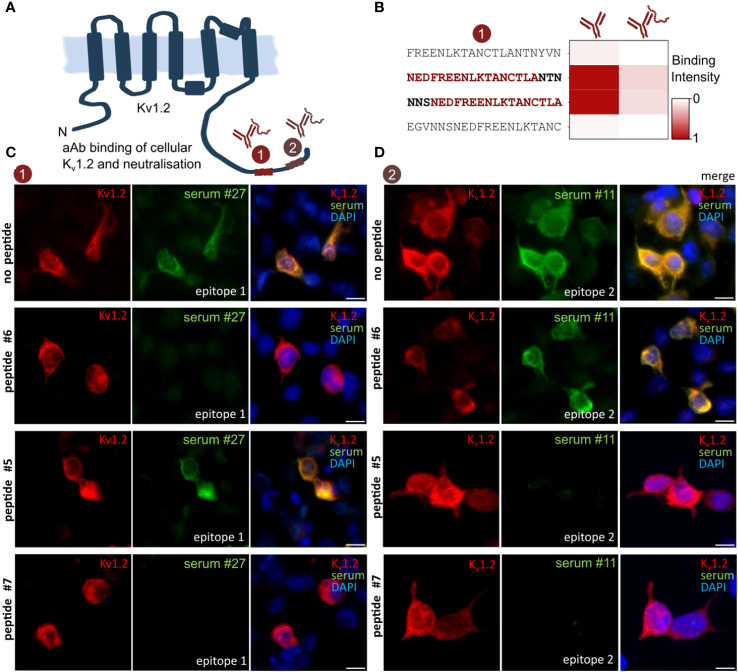
Anti–K_v_1.2 Autoantibody Neutralization excludes the presence of additional epitopes. **(A)** Scheme of the identified K_v_1.2 epitope landscape. **(B)** On-chip neutralization. Mapped peptides were synthesized and applied for pre-absorption experiments with patient sera. Upon incubation with peptide epitope, the microarray intensity has been neutralized. **(C)** HEK293 cell neutralization confirms E1 as solely mediator of the K_v_ binding. Transfected K_v_1.2 cells were permeabilized and incubated with either commercial anti-K_v_1.2 or patient sera, IgG binding was visualized with either anti-mouse IgG or anti-human. Peptides were designed based on the previous mappings (**Figures 1**, **2**). Pre-incubation with the mapped peptide epitope results in a complete autoantibody neutralization, whereas the non-neutralizing peptide epitope did not affect the binding. Neutralizing peptide #6 and #7: EDFREENLKTANCTLANTNY and ENLKTANCTLANTNYVNITK. Non-neutralizing peptide #5: ANCTLANTNYVNITKMLTDV. **(D)** HEK293 cell neutralization of E2 patient. Neutralizing peptide #5 and #7: ANCTLANTNYVNITKMLTDV and ENLKTANCTLANTNYVNITK. Non-neutralizing peptide #6: EDFREENLKTANCTLANTNY. Scale bar, 10 µm.

A commonly assumed limitation of array-based antibody screenings is the risk of missing autoantigen contributions from certain conformational and discontinuous epitopes not represented by the linear peptide display. In addition, anti-K_v_1.2 autoimmune response was previously observed to co-occur with unrelated neurological diseases (30, 31) as well as other K_v_1 complex–directed (7, 8) autoantibodies. To clarify the isoform specificity, K_v_1.1, K_v_1.2, and K_v_1.6 were expressed in HEK293 cells and probed against the serum IDs 1, 3, and 27 and then compared with the respective control antibodies (**Supplementary Figure 2**). In line with the deep mutational scans (**Figure 2**), K_v_1.1 and K_v_1.6 showed no binding, thus confirming K_v_1.2 specificity of the serum antibodies. To resolve contributions from possible additional antibodies binding via discontinuous epitopes that could not be resolved using peptide microarrays, we next conducted neutralization experiments of the identified epitope/autoantigen on chips and on cells. Soluble peptides were synthesized and purified on a preparative scale. On-chip neutralization resulted in a strongly reduced signal, thereby supporting a specific and exclusive binding through the identified epitopes toward K_v_1.2 in this format (**Figures 4A, B**). To search for additional, e.g., conformational, epitopes, we expressed K_v_1.2 in HEK293 cells (**Figures 4C, D**) and tested the neutralization of the observed immune response for the two identified epitopes and the commercial control antibody (**Figure 4C**). Transfected HEK293 cells were stained with serum pre-treated with neutralizing peptides, non-neutralizing peptides, and buffer only (**Figure 4C, D**). Patient’s sera were selected on the basis of their different epitopes for neutralization. Consequently, no residual binding was detected for either neutralized sample bearing E1 (**Figure 4C**) or E2 (**Figure 4D**). The non-neutralizing peptide had no impact on autoantibody binding. Notably, because no residual binding was detected, we conclude that K_v_1.2 autoimmunity is primarily mediated by autoantibodies that recognize the previously highlighted linear motif, without any contributions from additional linear or conformational epitopes.

### Peptide-based autoantibody neutralization on nerves and brain sections

**Figure 5:**
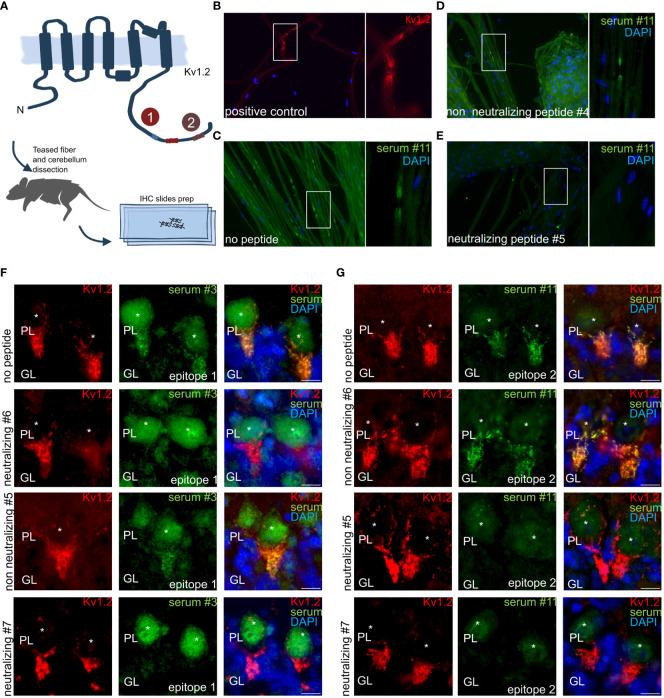
Anti–K_v_1.2 Autoantibody Neutralization Exclude the Presence of Co-Existing Peripheral and Central Autoantigens. **(A)** Teased fibers and mouse cerebellum preparation scheme. Teased fibers commercial Kv1.2 staining confirm correct antigen expression. Incubation of permeabilized mouse teased fibers with **(B)** clone K14/16 or **(C)** patient sera. **(D)** Non-neutralizing peptides did not interfere in teased fibers. **(E)** Pre-incubation with the mapped peptide epitope results in a complete autoantibody neutralization for patient 11. Non neutralizing and neutralizing peptides for E2: VNNSNEDFREENLKTANCTL, ANCTLANTNYVNITKMLTDV. **(F)** E1 epitope neutralization of patient sera immune response in mouse hippocampus. Neutralizing peptide #6, #7: EDFREENLKTANCTLANTNY, ENLKTANCTLANTNYVNITK. Non-neutralizing peptide #5: ANCTLANTNYVNITKMLTDV. **(G)** Neutralizing peptide leads to complete binding loss in mouse hippocampus for E2. Neutralizing peptide #5, #7: ANCTLANTNYVNITKMLTDV, ENLKTANCTLANTNYVNITK. Non-neutralizing peptide #6: EDFREENLKTANCTLANTNY. Neutralisation assay on mouse cerebellum: PL: Purkinje cell layer, GL: granular layer. *indicates Purkinje cell soma. Scale bar, 10 µm.

Prompted by the confirmation of the identified epitopes as sole driver of the observed immune response in transfected recombinant cells, we next explored autoantibody binding toward the native autoantigens in their cellular context. Here, we applied the neutralized sera on teased fibers (**Figure 5A**). Commercial antibody (K14/16) signals recapitulate the expected juxtaparanodal binding on teased fibers (**Figure 5B**). Sera applied together without peptide (**Figure 5C**) and with non-neutralizing peptide (**Figure 5D**) recapitulated the same labeling. In stark contrast, sera pre-treated with neutralizing peptide display a complete loss of binding signal (**Figure 5E**). Thus, complete anti-K_v_1.2 epitope-specific neutralization was achieved using only the minimal K_v_1.2 epitope. Remarkably, no residual autoantibody binding was detected in the nerve tissues, and the tested sera did not cross-react with additional autoantigens co-expressed peripherally. This corroborates the hypothesis of selective K_v_1.2 autoimmunity without co-existing peripheral autoantibodies, thus contrasting previous observations (7, 8, 30, 31).

The neutralization in peripheral nerves was complemented by neutralization in tissue slices from the central nervous system, specifically mouse cerebellum, where K_v_1.2 expression is high at the axon initial segment (AIS) of Purkinje cells. MAb K14/16 served as a K_v_1.2 antibody control for specific binding of autoantibodies. Here, the typical axonal initial segment stainings were observed on the Purkinje cell layer. E1 (**Figure 5F**) and E2 (**Figure 5G**) positive serum was applied in presence of several neutralizing and non-neutralizing peptide variants.

Taken together, the neutralization data in cell and tissues confirmed the high selectivity for the identified peptide epitopes, showing no detectable residual binding. Thus, leading to the exciting conclusion that a single, broadly shared epitope may contribute significantly to the often-reported K_v_1 autoimmunity (7, 8, 30, 31), in some cases, even without coexisting autoantigens or other conformational epitopes. In addition, the disease association of the detection of the here defined immunodominant epitope in CSF suggests implications for diagnosis, possibly even the pathology of a subgroup of autoimmune neuropsychiatric phenotypes.

## Conclusion

Among K_v_1 complex–directed autoantibodies, anti-K_v_1 are among the most prevalent (7, 8); compared with LGI1 and CASPAR2 autoantibodies subgroups, their association with clinical syndromes and their immunotherapy responsiveness, however, appears less clear. Despite the association of K_v_1 subfamily autoantibodies to neurological autoimmune diseases (5, 6, 9) and their pathology (7, 8, 10, 11, 20–22) and their resulting diagnostic and therapeutic potential the involved K_v_1 epitopes remained largely undefined. Here, we provide a first K_v_1.2 autoantibody epitope landscape within a cohort of 36 K_v_1.2-exclusive neuropsychiatric patients and 18 healthy controls. In contrast to structural (24) and recombinant protein-based approaches (32–34), the array approach (2–4) combined with cell-based and tissue-based studies enabled the high-throughput molecular characterization of the autoantibodies directly from patient samples. Our data depict an unexpectedly monospecific and uniform autoantibody repertoire with two shared responses including one immunodominant K_v_1.2 and K_v_1.3 autoantibody epitope common to most of the patients tested here. Binding to additional subfamily members K_v_1.1, K_v_1.4, and K_v_1.6 has been excluded by array or cellular assays. Moreover, the notable similarity in binding responses and the preservation of the required residues between patients implies a shared molecular genesis, which may include viral or bacterial antigens. In line with a possibly elevated immunogenic potential, K_v_ positivity was reported in swine abattoir workers negative for both anti-LGI1 and anti-CASPR2 (35) as well as co-occurring with non-immunogenic neurological disease (30, 31). Sequences with high similarity to “EENLKTANCTLANTNYVNITK,” namely, “EESLKTGNAG” and “ANTIYVNITKMLT,” were previously reported (36, 37) as highly immunogenic. Autoantibody response is exclusively directed against K_v_1.2 as substantiated by juxtaparanodal reactivity on teased nerves and AIS labeling in Purkinje cells. Importantly, the mapped epitopes enabled the complete neutralization of the observed reactivity, thereby establishing the outlined auto-antigen region as the primary mediator and even sole driver, of the observed autoimmune response. It remains to be seen whether these antibodies can bind *in vivo* (23, 24) to interfere with Kv channel function or protein-protein interactions such as the scaffolds PSD-95 and Cortactin, which are reported to bind near the N-terminal "PQTP" (38) or C-terminal "TDV" (39). On the other hand, a direct functional effect on the K_v_1.2 channel has been shown to exacerbate a pro-epileptic state (21) and could potentially modulate the excitability of entire complex (40–42). The high prevalence of the here identified K_v_1 epitope argues for future detailed studies on a possible intracellular action or indirect mechanisms such as T-cell cytotoxicity (43). Investigation of the relevant T-cell subpopulation involved and the HLA association, together with a possible tolerance mechanism, could shed light on the identified disease-specific antigen and why it is shared by several patients, similar to multiple sclerosis (44). CSF was previously reported to harbor enhanced diagnostic value in autoimmune neurological diseases (45, 46), but detection of low autoantibody titers remains challenging for conventional ELISA (enzyme-linked immunosorbent assay) and cell-based assay approaches that are limited in the density of the antigen display and further require the successful expression and immobilization of the antigens. Notably, the here reported prevalent and immunodominant K_v_1 epitope achieves sensitivity and specificity for autoantibody detection in CSF. Significantly, the detected presence of autoantibodies in CSF associates with the clinical symptoms of cognitive impairment, thus highlighting its value for K_v_1.2 autoantibody confirmation. This study provides a prevalent and immunodominant epitope together with the underlying autoantibody binding requirements in K_v_1 autoimmunity in neuropsychiatric patients. Thus, setting the stage for future investigation of the molecular origin and a potential intracellular action of the here identified mono-specific K_v_1 antibodies. These studies may focus on the analysis of clinical presentations, longitudinal samples, and immunotherapy responsiveness. Finally, the reported epitope also provides a means for isolating or even depleting the potentially disease-defining autoantibodies or their respective B cells.

In summary, our study defines an immunodominant epitope as single determinant in K_v_1 autoimmunity and thus outstanding diagnostic potential.

## Data availability statement

The original contributions presented in the study are included this articles/**Supplementary Material**. Further inquiries can be directed to the corresponding authors.

## Ethics statement

The studies involving humans were approved by Ethics Committee of Charité University Medicine Berlin (EA1/258/18). The studies were conducted in accordance with the local legislation and institutional requirements. The participants provided their written informed consent to participate in this study. Ethical approval was not required for the studies on animals in accordance with the local legislation and institutional requirements because only commercially available established cell lines were used.

## Author contributions

IT: Data curation, Formal analysis, Investigation, Methodology, Visualization, Writing – original draft. FA: Data curation, Formal analysis, Investigation, Methodology, Writing – review & editing. KK: Investigation, Methodology, Writing – review & editing. MN: Investigation, Writing – review & editing. A-LW:Data curation, Formal analysis, Investigation, Visualization, Writing – review & editing. RM: Conceptualization, Formal analysis, Investigation, Methodology, Project administration, Resources, Validation, Writing – review & editing. SM: Formal analysis, Investigation, Methodology, Writing – review & editing. ID: Formal analysis, Investigation, Methodology, Writing – review & editing. MM: Investigation, Writing – review & editing. MB: Investigation, Writing – review & editing. PC: Data curation, Formal analysis, Investigation, Methodology, Resources, Supervision, Validation, Writing – review & editing. IA: Formal analysis, Investigation, Resources, Writing – review & editing. AK: Formal analysis, Investigation, Methodology, Writing – review & editing. ME: Data curation, Formal analysis, Investigation, Methodology, Resources, Supervision, Writing – review & editing. LK: Formal analysis, Investigation, Methodology, Writing – review & editing. CV: Data curation, Funding acquisition, Supervision, Resources, Validation, Writing – review & editing. KD: Conceptualization, Data curation, Funding acquisition, Investigation, Methodology, Project administration, Resources, Supervision, Writing – review & editing. HP: Conceptualization, Data curation, Funding acquisition, Project administration, Resources, Supervision, Validation, Writing – original draft. HM: Conceptualization, Data curation, Funding acquisition, Project administration, Resources, Supervision, Validation, Visualization, Writing – original draft.

## Supplementary material

Supplementary Figure 1 Anti-K_v_1.2 detection in three sera from healthy individuals highlights the presence of “naturally” occurring autoantibodies **(A)** Four additional samples (N, O, P, Q) coming from the EUROIMMUN screening were subjected to microarray analysis. Three sera resulted positive for E1; **(B)** subsequently, these samples were fully profiled. Here, the characteristic E1 fingerprint was recapitulated.

Supplementary Figure 2 Binding of serum samples #1, #3, #27, healthy control (HC) serum and a commercial antibody against the according K_v_ channels to transfected HEK293 cells. Cells were transfected with either human K_v_1.1 **(A)**, K_v_1.2 **(B)** or K_v_1.6 **(C)** and stained with commercial antibodies (red). Binding of patient serum was visualized with anti-human-IgG-Cy3 antibody (green). Scale bar refers to 10 µm.
