# Supplementary figures and images for: Molecular dissection of an immunodominant epitope in Kv1.2-exclusive autoimmunity
Source: Front Immunol. 2024 Apr 11;15:1329013. doi: 10.3389/fimmu.2024.1329013 (PMC11043588; doi:10.3389/fimmu.2024.1329013)

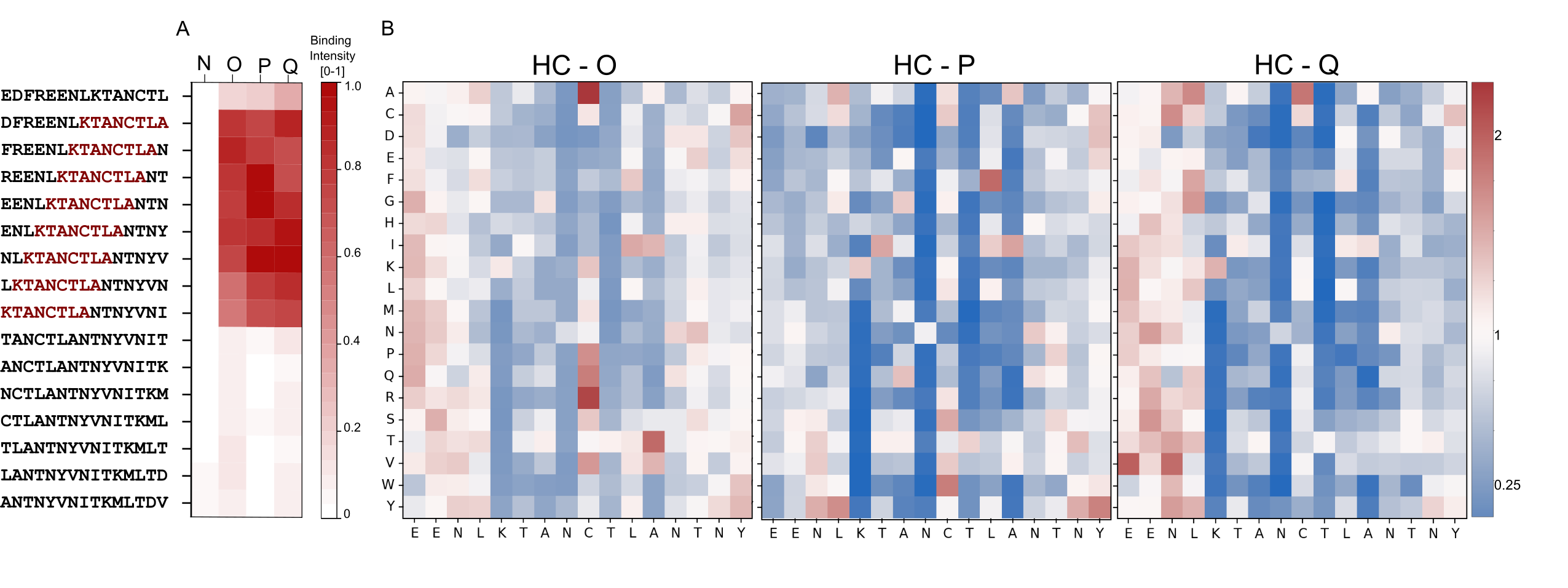

Supplement: Supplementary Figure 1 — Anti-Kv1.2 detection in three sera from healthy individuals highlights the presence of “naturally” occurring autoantibodies (A) Four additional samples (N, O, P, Q) coming from the EUROIMMUN screening were subjected to microarray analysis. Three sera resulted positive for E1; (B) subsequently, these samples were fully profiled. Here, the characteristic E1 fingerprint was recapitulated. [file Image_1.tiff]

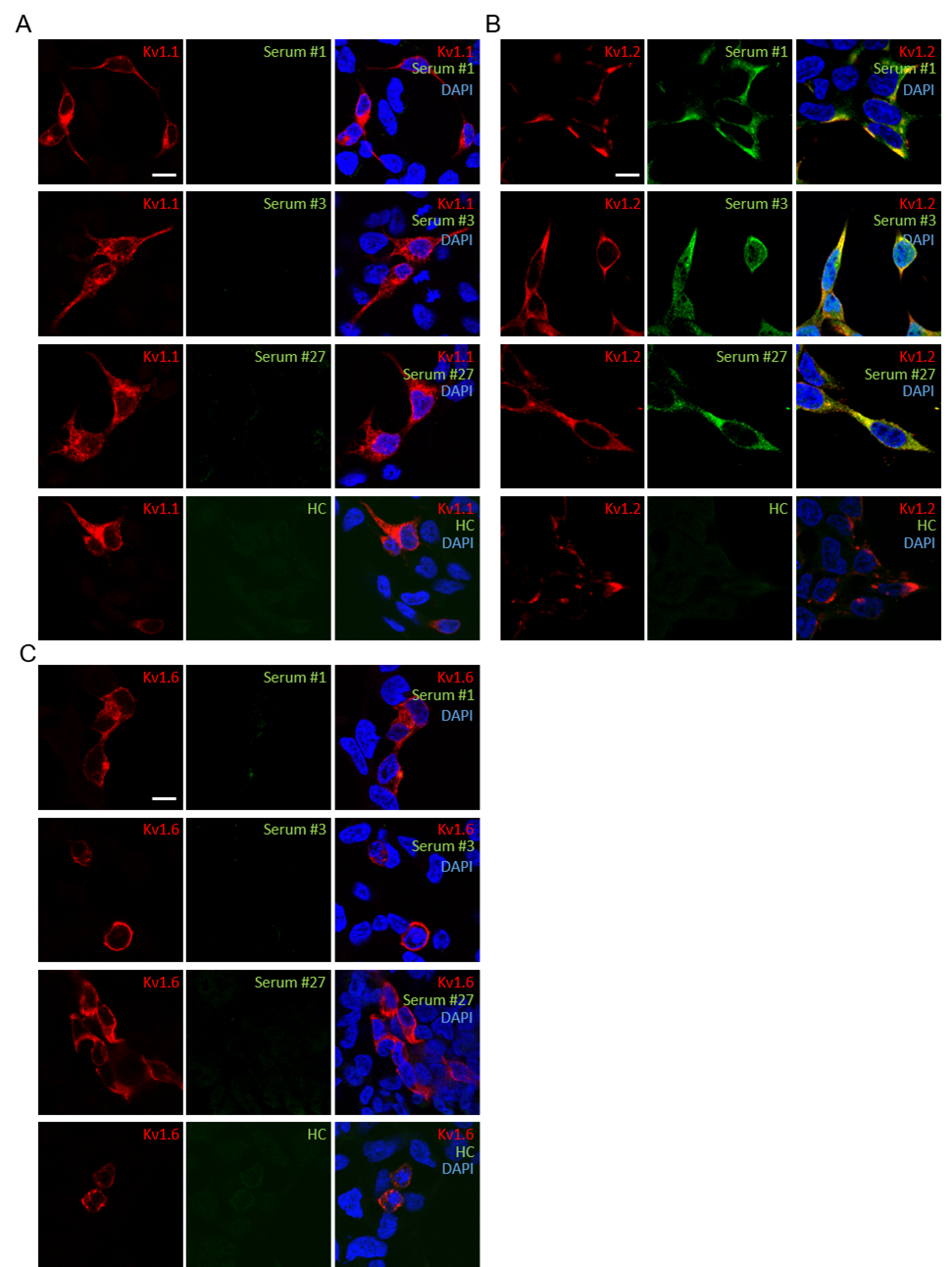

Supplement: Supplementary Figure 2 — Binding of serum samples #1, #3, #27, healthy control (HC) serum and a commercial antibody against the according Kv channels to transfected HEK293 cells. Cells were transfected with either human Kv1.1 (A), Kv1.2 (B) or Kv1.6 (C) and stained with commercial antibodies (red). Binding of patient serum was visualized with anti-human-IgG-Cy3 antibody (green). Scale bar refers to 10 µm. [file Image_2.tif]
